# Central nervous system regulation of diffuse glioma growth and invasion: from single unit physiology to circuit remodeling

**DOI:** 10.1007/s11060-024-04719-x

**Published:** 2024-06-04

**Authors:** Thiebaud Picart, Shawn Hervey-Jumper

**Affiliations:** 1 Department of Neurological Surgery, University of California, San Francisco, San Francisco, CA USA; 2 Department of Neurosurgery, Hospices Civils de Lyon, Bron, France; 3 Weill Institute for Neurosciences, University of California San Francisco, San Francisco, CA USA

**Keywords:** Cancer Neurosciences, Circuit Remodeling, Glioblastoma, Malignant glioma, Neuroplasticity

## Abstract

**Purpose:**

Understanding the complex bidirectional interactions between neurons and glioma cells could help to identify new therapeutic targets. Herein, the techniques and application of novel neuroscience tools implemented to study the complex interactions between brain and malignant gliomas, their results, and the potential therapeutic opportunities were reviewed.

**Methods:**

Literature search was performed on PubMed between 2001 and 2023 using the keywords “glioma”, “glioblastoma”, “circuit remodeling”, “plasticity”, “neuron networks” and “cortical networks”. Studies including grade 2 to 4 gliomas, diffuse midline gliomas, and diffuse intrinsic pontine gliomas were considered.

**Results:**

Glioma cells are connected through tumour microtubes and form a highly connected network within which pacemaker cells drive tumorigenesis. Unconnected cells have increased invasion capabilities. Glioma cells are also synaptically integrated within neural circuitry. Neurons promote tumour growth via paracrine and direct electrochemical mechanisms, including glutamatergic AMPA-receptors. Increased glutamate release in the tumor microenvironment and loss of peritumoral GABAergic inhibitory interneurons result in network hyperexcitability and secondary epilepsy. Functional imaging, local field potentials and subcortical mapping, performed in awake patients, have defined patterns of malignant circuit remodeling. Glioma-induced remodeling is frequent in language and even motor cortical networks, depending on tumour biological parameters, and influences functional outcomes.

**Conclusion:**

These data offer new insights into glioma tumorigenesis. Future work will be needed to understand how tumor intrinsic molecular drivers influence neuron-glioma interactions but also to integrate these results to design new therapeutic options for patients.

## Introduction

Malignant gliomas are the most frequent malignant primary brain tumours in adults and are associated with a poor prognosis despite an improved understanding of tumor intrinsic drives of disease progression [1, 2]. Surgical resection, temozolomide with brain irradiation and, TTF-fields have been demonstrated to significantly prolong overall survival, yet recurrence occurs for nearly all patients and few patients survive longer than 2 years [3]. Nervous system regulation of cancer (termed Cancer Neuroscience) is an emerging discipline that focuses on defining and therapeutically targeting interactions between the nervous system and cancer [4]. There is a growing body of evidence defining the complex bidirectional interactions between neurons and malignant glial cells [4]. The central nervous system in adults maintains normal function supporting cognitive operations through cellular level neuron-to-neuron and neuron-to-glia interactions, which collectively establish local synaptic circuits and distributive cognitive networks. Therefore, while adaptive neural plasticity maintains healthy circuit dynamics through synaptic and cellular mechanisms of plasticity, these same processes may influence glioma behavior. Emerging evidence in this field may lead to new therapeutic strategies for patients. Experimental models of disease range from single cell electrophysiology of neoplastic cells and neurons to invasive and non-invasive neuronal circuit interrogation. Reviews to date on this topic focus predominantly on cellular level evidence. The aim of this study is to highlight the evidence supporting activity dependent mechanisms of glioma proliferation including both cellular and synaptic network level investigations.

## Methods

Literature search was performed on the Medline electronic database between 2001 and 2023. The keywords used were “glioma”, “glioblastoma”, “circuit remodeling”, “plasticity”, “neuron networks” and “cortical networks”. The references of selected articles were also examined to identify additional studies and included studies focused on WHO grades 2–4, diffuse midline gliomas, and diffuse intrinsic pontine gliomas.

## Results

### Cellular level evidence of neuronal regulation of glioma proliferation and invasion

**Fig. 1:**
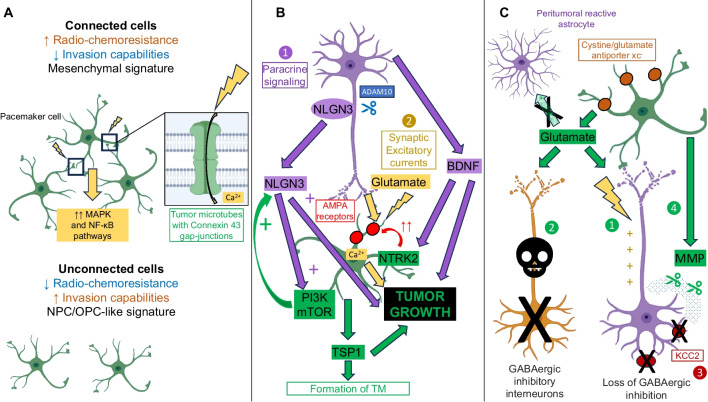
Simplified representation of the interactions between tumours cells and the bidirectional crosstalk between neurons and tumour cells (created with Biorender). **A.** Interactions between tumour cells. Tumor cells (green) form an extensive network, mediated by tumor microtubes that bear Connexin 43 gap-junctions, and are resistant to radio-chemotherapy. Within the network, pacemaker-like cells, thanks to KCa 3.1 channels (yellow), display rhythmic Ca^2+^ oscillations that are transmitted to the network and that drive tumour aggressiveness via activation of the MAPK and NF-κB pathways. Unconnected cells have increased invasion abilities. **B.** Neuronal activity-regulated tumour growth. First, neurons (purple) emit a paracrine signaling, notably mediated by BDNF and NLGN3, that stimulates tumour growth. NLGN3 is clived from neural cells by the ADAM10 sheddase. NLGN3 activates the PI3K-mTOR pathway and feedforwards its expression. Second, through excitatory glutamate neuro-gliomal synapses, whose establishment is eased by NLGN3, neurons activity stimulates tumors growth. BDNF binding to NTRK2 receptors increases the expression of AMPA receptors (red), which strengthen glutamate signaling. The neuron-dependent secretion of TSP1 by tumour cells contributes to tumour microtube formation and glioma progression. **C.** Glioma-induced neuronal activity modifications. Glutamate is released by glioma cells through the cystine/glutamate antiporter xc^−^ (brown). Peritumoral reactive astrocytes have a decreased capability to uptake glutamate. First, the increased glutamate rate in the tumour microenvironment induces neuronal hyperexcitability. Second, glutamate-toxicity leads to the death of fast-spiking GABAergic inhibitory neurons. Third, the drop in the neuronal expression of the potassium/chloride transporter KCC2 (dark red) is responsible for the switch from inhibitory to excitatory of GABA signaling. Fourth, the disruption of perineuronal nets by glioma-secreted matrix metalloproteinases amplifies the loss of GABAergic inhibition and glutamate-induced neuronal death

In vitro and in vivo models offer the opportunity to understand the complex bidirectional interactions between neurons and malignant glial cells, detailed in Fig. 1, within the tumour microenvironment, at the cellular and molecular levels (Table 1).

**Table 1:** Principal techniques and neuroscience tools used to study glioma-induced malignant circuit remodeling at the microscopic level

Technique	Principle	Main results
In vivo multiphoton laser-scanning microscopy	Following of growing tumors transduced with lentiviral vectors for multicolour imaging, in the mouse brain (under anesthesia), thanks to a custom-made aperture, for up to one year. For angiograms, fluorescent dextranes are injected in the tail vein. The following of calcium indicators transduced with lentiviral vectors is also feasible	Discovery of TM [5] Discovery of pacemaker cells within the glioma cell network [6] Description of the behavior of tumour cells [7–9]
In vivo optogenetic control of cortical neuronal activity	Light stimulation of the peritumoral cortex through an optical-neural interface placed just below the pial surface, in a mouse model expressing the excitatory opsin channelrhodopsin-2 (ChR2) in deep cortical projection neurons	Discovery of neuronal activity-regulated glioma growth [10–12]
Tumour neurons coculture	In vitro culture of tumor cells in the presence of neurons	Discovery of the role of TSP1 [13]
Patch-clamp test	Electrophysiological recordings focusing on isolated cells or tissue sections, using a microelectrode, in a recording chamber, at rest or after various stimulations	Discovery of the synaptic and electrical integration of glioma into neural circuits [14, 15]
Electophysiological recording on brain slices taken from xenograft models	Exposition of slices taken in xenograft mouse models to different conditions, notably in a pro-epileptogenic medium lacking magnesium, in a recording chamber. Of note, spectroscopic analysis is also possible to assess tissue secretion under defined conditions	Cortical hyperexcitability within peritumoral area [16–20] Demonstration of glutamate release by tumour cells [16]

#### Glioma-glioma networks promote glioma growth and treatment resistance

Malignant astrocytoma mouse xenografted tumors analyzed with in vivo multiphoton laser-scanning microscopy uncovered the presence of Tumour Microtubes (TM), that correspond to ultra-long membrane protrusions, stabilized by p120 catenin. Through TM, glioma cells form an extensive and non-randomly organized network mediated by Connexin 43 gap junctions [21] and exchange ions and molecules. The neuronal growth-associated protein GAP-43 conditions TM formation and function and, in concert with the membrane protein linked to neuronal development TTYH1, drives TM-mediated invasion properties and proliferation [5, 6, 22, 23]. TM also establish connections between tumor cells and non-tumoral astrocytes [7]. NOTCH1 regulates network connectivity and its downregulation induces TM extension [8]. A subpopulation of highly connected pacemaker-like glioma cells display rhythmic Ca^2+^ oscillations, relying on the calcium-dependent potassic channel KCa3.1. These Ca^2+^ oscillations are transmitted to the network and activate the frequency-dependent MAPK and NF-κB pathways [6, 24]. TM are involved in treatment failures. First, they ease repopulation of the surgical cavity after glioma resection [9]. Second, connected cells are less susceptible to radiation-induced [5] and chemotherapy-induced cytotoxicity than unconnected cells [9], but have however decreased invasion abilities [5, 7, 8, 23]. Connected and unconnected cells express a mesenchymal-like and a neural/oligodendroglial precursor-like signature, respectively [7].

Pharmacological inhibition of Connexin 43 or gap junction but also meclofenamate inhibition of intercellular cytosolic traffic via gap junctions reduced glioblastoma cell resistance to Temozolomide and Lomustine, independently of MGMT status [25–27]. Consistently, the genetic inactivation of Connexin 43 or GAP-43 and the senicapoc pharmacological inhibitor of potassic channel KCa3.1 slowed down xenograft progression in mice [5, 6, 9]. *TTYH1* knockdown decreased the rate of invasive tumour cells harboring one or two TM but not the rate of hyperconnected cells harboring more than four TM, unveiling a functional and molecular heterogeneity among TM [23]. Consequently, TM formation and function could offer new therapeutic targets.

#### Neuronal activity drives malignant glioma growth

Through the use of in vivo optogenetic control of cortical neuronal activity in patient-derived pediatric glioblastoma xenograft models in mice expressing the excitatory opsin channelrhodopsin-2, it was demonstrated that neurons stimulate glioma growth through paracrine signaling mediated by Brain-Derived Neurotrophic Factor (BDNF) and the soluble synaptic adhesion protein neuroligin-3 (NLGN3) [10–12]. NLGN3 promotes tumour growth and feedforwards its own expression in glioma cells, through induction of the PI3K-mTOR pathway activity. Accordingly, NLGN3 expression is inversely correlated with overall survival [11] and conditions the growth of many subtypes of pediatric and adult gliomas [12]. NLGN3 is cleaved from neurons and oligodendrocytes precursor cells by the ADAM10 shedddase. ADAM10 inhibition reduces xenograft growth by preventing NLGN3 release into the tumour microenvironment and could represent the basis of new therapeutic strategies [12]. The relevance of INCB7839, an ADAM 10 and 17 inhibitor, for the treatment of pediatric high-grade gliomas is assessed in an ongoing randomized clinical trial (NTC04295759).

NLGN3 paracrine signaling upregulates the expression of several synapse-related genes [12], and promotes the establishment of aberrant synapses, localized on TM. The electrochemical communications between presynaptic neurons and postsynaptic malignant glial cells is mediated by glutamatergic α-amino-3-hydroxy-5-methyl-4-isoxazole propionic acid receptors (AMPARs) [10, 14]. AMPAR trafficking to the glioma cell membrane is promoted by docking of BDNF to the receptor NTRK2, resulting in an increased amplitude of glutamate-evoked currents in the malignant cells. Pharmacological or genetical inhibition of NTRK2 decreases synaptogenic mechanisms and prolongs survival in xenograft models [15]. A high-neural epigenetic signature associated to overexpression of synaptic genes was consistently correlated with increased connectivity according to functional Magnetic Resonance Imaging (fMRI) and Magneto-Electro-Encephalography (MEG) [28]. Additionally, the tumor cell subpopulation which overexpresses synaptic genes is mainly of oligodendroglial or neural progenitor-like signature [10, 28, 29] but the link between synaptic transmission and stemness phenotype still remains to be elucidated.

Neuronal activity evokes excitatory post-synaptic currents but also non-synaptic activity-dependent currents, respectively calcium- and potassium-mediated, that are amplified by gap junction-mediated tumour interconnections through TM, forming an electrically coupled network [10, 14]. Synaptic and electrical integration of glioma into neural circuits, and glutamate binding to AMPARs favor tumour progression. Indeed, neuronal activity drives TM dynamics and increases the number of TM branching events via calcium signaling, but also the invasion speed of unconnected glioma cells via transient neuro-gliomal synapses with AMPARs [7, 14, 30]. Moreover, depolarization of glioma cell membranes promotes proliferation [10]. Consistently, the occurrence of gliomas is higher in brain regions which display higher intrinsic activity levels according to MEG [31]. Furthermore, high-functionally connected tumour regions are enriched in a tumor cell subpopulation with synaptogenic properties, which develops TM and proliferates on the presence of neurons, under the dependence of glioma-secreted Thrombospondin 1 (TSP1). Interestingly, the presence of high-functional connectivity areas within tumour negatively influence cognitive and survival prognosis [13]. However, remote activity-dependent glioma progression mechanisms were recently identified. They are driven by an infiltrating cell population highly-expressing axon guidance genes, notably SEMA4F, and whose activity depends on contralateral callosal projection neurons [32].

Cytostatic effects are observed following pharmacological electrochemical signaling blockade [33], using the AMPAR-blocking drug perampanel, meclofenamate [10, 14] or using gabapentin which both inhibit TSP1 and the branched-chain amino acid aminotransferase (BCAT1) thus lowering extracellular glutamate level [13, 34]. The anti-tumoral effects of glutamate signaling inhibitors, including gabapentin, meclofenamate and perampanel, are assessed in several on-going trials [16, 35, 36].

#### Malignant glioma proliferation promotes neuronal hyperexcitability

Electroencephalograms performed in glioma murine models demonstrate spontaneous and recurring abnormal events, compatible with progressive epileptic activity, with a concurrent enrichment in a tumor cell population expressing synaptic genes, suggesting propagation of synapse associated genes [17]. Cortical slices following malignant glioma implantation demonstrate increased glutamate release from the tumor mass, mediated by the cystine/glutamate antiporter xc^−^ [17, 18, 30], whose expression is anticorrelated to this of p53 [19]. Moreover, peritumoral reactive astrocytes have a decreased ability to uptake glutamate and potassium. Some peritumoral astrocytes also display a depolarized resting membrane potential, further contributing to alter potassium and glutamate homeostasis [20]. A resultant glutamatergic epileptiform hyperexcitability spreading into peritumoral areas was identified by extracellular field recordings at sites distant from glioma [17], consistently with the results of human intraoperative ECoG recordings [10]. Extracellular field recordings performed on brain slices taken from xenograft models revealed that the onset latency of magnesium-free-induced epileptiform activity was shorter than in healthy slices. Moreover, the incidence of ictal-like events was higher. Blockade of the cystine/glutamate antiporter xc^−^ and thus glutamate release from the tumour mass using sulfasalazine decreased hyperexcitability and was able to reduce not only the frequency of epileptic events in tumour-bearing mice [17] but also neuronal activity-regulated glioma growth [37].

Independently, peritumoral cortex taken from mice orthotopic xenografts also displays a distance-dependent loss of parvalbumin-positive fast-spiking GABAergic inhibitory interneurons, attributable to glutamate neurotoxicity, and a reduced neuronal expression of the potassium/chloride plasmalemmal transporter KCC2, changing response to GABA from inhibitory to excitatory potentials [33, 38, 39]. Additionally, perineuronal nets, which represent a complex lattice-like extracellular matrix, acting as an electrostatic insulator that reduces specific membrane capacitance, are degraded in a distance-dependent manner by glioma-released matrix metalloproteinases. This mechanism amplifies the loss of GABAergic inhibition but also glutamate-induced neuronal death [39].

Taken together, these results demonstrate that hyperexcitability results from peritumoral synaptic network disruption in the setting of malignant gliomas [20, 37, 38] and suggest new therapeutic targets for controlling peritumoral hyperexcitability. Interestingly, in a mouse model, it was evidenced that some hotspot-mutations of *PI3KCA* induced increased network hyperexcitability compared to others. Notably, neurons adjacent to C420R- and H1047R-mutant gliomas displayed enhanced synaptic imbalance, which was attributed to the secretion of glypican 3 in the case of C420R variant [40], bridging genetics with peritumoral synaptic remodeling.

### Network level evidence of nervous system regulation of glioma proliferation and invasion

**Fig. 2:**
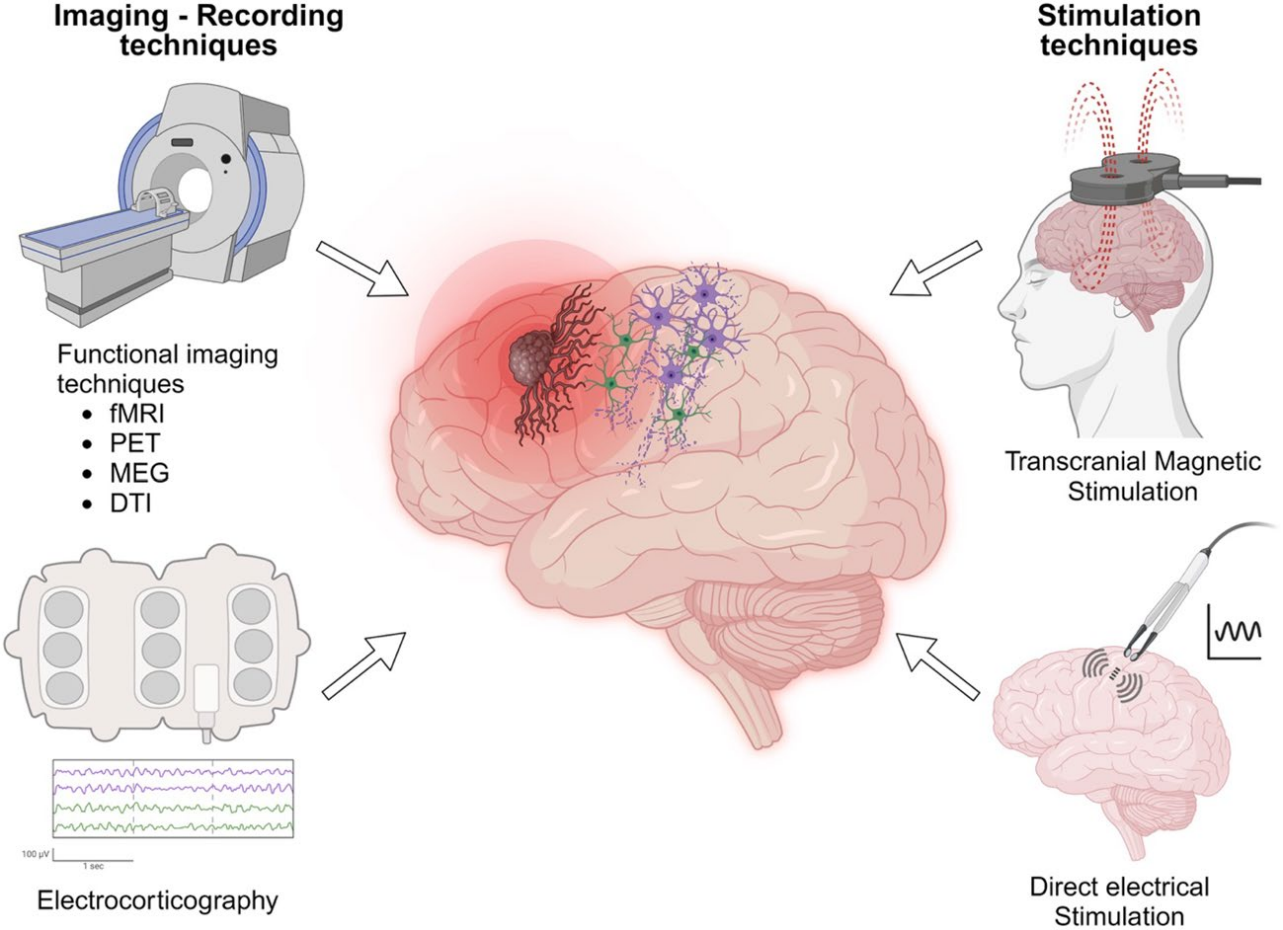
Representation of the different tools offering the possibility to study glioma-induced malignant circuit remodeling as well as nervous system regulation of glioma proliferation and invasion at the macroscopic level (created with Biorender)

Resting state and task-based investigations are available to indirectly and macroscopically probe functional plasticity mechanisms and circuit remodeling caused by malignant gliomas (detailed in Fig. 2 and Table 2). Network level understanding of glioma development has recently come into clearer view because of emerging technologies.

**Table 2:** Principles and characteristics of the different techniques dedicated to study glioma-induced malignant circuit remodeling at the macroscopic level

	Principle	Assessment of plasticity mechanisms	Invasive	Identification of whole-brain remodeling	Advantages	Limitations*
Functional imaging techniques	fMRI: Detection of the BOLD (blood-oxygen- level-dependent) effect during tasks or at resting state	1. Comparison with healthy volunteers 2. Longitudinal comparison of preoperative and postoperative imaging 3.Comparison of the two hemispheres (lesional vs unaffected) in the same patient	No	Yes	Routinely used for motor and language mapping, with high specificity and sensitivity Good anatomical resolution	Unable to discriminate compensable and not compensable functional areas Reliability can be impaired by neuro-vascular uncoupling in high-grade gliomas Possible inconsistence with direct electrostimulations
	PET: Detection of regional cerebral blood flow/metabolic changes during tasks				-	Irradiant Unable to discriminate compensable and not compensable functional areas
	MEG: Detection of intrinsic magnetic fields produced during tasks or at resting state				High spatial and temporal resolution Not impaired by neuro-vascular uncoupling	Unable to discriminate compensable and not compensable functional areas Few availability Expensive
	DTI: Reconstruction of white-matter tracts based on diffusion parameters				Routinely used for fiber tracking with a good sensitivity	Reliability depending on the chosen settings Impaired resolving in areas where fiber cross
Stimulation techniques	rTMS: Application of magnetic pulses thanks to a coil in order to disrupt cortical activity	1. Comparison with healthy volunteers 2. Longitudinal comparison of preoperative and postoperative acquisitions	No	No	High sensibility	Limited specificity Lack of standardization regarding stimulation parameters Risk of seizures, pain and incomfort
	DES: direct electrical stimulations leading to a patient-specific cortical and subcortical mapping, prior to and during resection of gliomas located in eloquent areas in awake condition (or asleep for motor testing)	Longitudinal comparison in patients who undergo at least two glioma resections	Yes	No	Considered as the gold-standand for the identification of eloquent areas (high specificity, high spatial resolution and real-time feedback) Assessment of both cortical and subcortical plasticity	Possible intraoperative seizures Requires patient intraoperative participation
ECoG	Intraoperative subdural recording of the cortical activity	Comparison of infiltrated-appearing and healthy-appearing cortex in the same patient	Yes	No	-	-

^*^ Independently of the technique, in case of longitudinal comparison in the same patient, identified modifications may not only result from glioma-induced circuit remodeling but also from surgery and medical treatments possibly administered after tumour resection

#### Functional MRI (fMRI)

Four different glioma-induced remodeling patterns in patients have been identified using fMRI with the appearance of additional activation sites within (1) the tumor, (2) the ipsilateral peritumoral cortex, (3) the distant ipsilateral cortex, and (4) the contralateral normal appearing cortex [41]. Indeed, in a series of patients with gliomas located at an average distance of 3.9 ± 3.5 cm from the hand motor region, 62% and 46% of patients exhibited an ipsilateral and contralateral recruitment, respectively. Ipsilateral recruitment decreased as tumour volume increased and distance from primary motor cortex decreased and vice versa [42]. However, patients with gliomas located in or near motor areas, but without any motor deficit, had a significant reduction in inter-hemispheric functional connectivity between bilateral primary motor cortices, compared to age-matched healthy controls [43]. Regarding the language network, right-handed patients with a left hemisphere glioma also had a global reduction of bilateral functional connectivity, compared to healthy controls. The most affected node was the left temporo-parietal junction [44].

According to resting-state fMRI, compared to healthy controls, patients with glioma had a decreased functional connectivity concerning the whole-brain, and not restricted to the lesional hemisphere [45, 46]. The importance of these alterations was correlated with high tumor grade, negative IDH status and decreased neuropsychological performances but not with tumour size or location [45]. Besides, Default Mode Network connectivity was modified in patients managed for left-sided gliomas, with increased and decreased integration in hippocampal and prefrontal areas, respectively [47]. Finally, a higher intra-network functional connectivity strength within glioblastoma was found to be independent of tumour size but predicted a better overall survival [48].

#### Positron Emission Tomography (PET)

One of the first studies demonstrating remodeled network connectivity caused by chronic disease of the central nervous system was identified using PET imaging of amyloid beta in patients with Alzheimer’s disease [49]. PET of gliomas located in the hand motor region showed that, compared to the unaffected side, the activations were shifted by 20 ± 13 mm (SD), either along the mediolateral body representation of motor cortex or into premotor or parietal somatosensory cortex [50]. Additional activation of the supplementary motor area was occasionally present [50]. Regarding the plasticity of the language network, two compensatory mechanisms, whose occurrence depended on tumour location, were identified. First, at the intra-hemispheric level, left fronto-lateral regions other than classical language areas can be recruited. Second, at the inter-hemispheric level, fronto-lateral activation can appear in the right nondominant hemisphere, especially in patients with frontal or temporal posterior tumours, possibly representing a loss of transcallosal collateral inhibition [51].

#### MagnetoEncephaloGraphy (MEG)

The comparison of whole-brain activation motor maps performed in the same patients at initial diagnosis and compared with first recurrence has demonstrated a shift in activation peaks in the ipsilateral and contralateral motor cortices [52]. Specifically, motor activity following glioma progression is associated with contralesional hemisphere activation for speech and motor tasks. Tumor location, presence of a motor impairment and longer time lapse were associated with greater cortical remodeling [52]. The same methodology applied to language mapping highlighted a shift in language laterality index in about 30% of patients. Magnitude and relative direction of the shift depended on tumor location and initial language dominance, as shift was greater in patients with increased lateralization compared to those with bilateral representation [53]. However, these studies not only reflected glioma-induced but also surgical-induced plasticity mechanisms.

#### Electrocorticography (ECoG)

In awake patients with malignant gliomas, at resting state and during speech, an increased high-gamma band range power is detectable outside of the necrotic tumour core, in glioma-infiltrated brain compared to healthy-appearing brain, consistent with cortical hyperexcitability [10, 13]. Furthermore, intraoperative subdural local field potentials using ECoG recordings before awake resection of dominant hemisphere malignant gliomas proved that glioma-infiltrated cortex is able to engage in synchronous activity during task performance, similarly to normal-appearing cortex, but recruits a widened spatial network [13, 54], as observed with imaging techniques [51]. Glioma-infiltrated cortex has decreased entropy compared to normal-appearing cortex and may therefore be less efficient to encode information during nuanced tasks as production of monosyllabic and polysyllabic words. Consistently, in glioma-infiltered cortex, signals corresponding to monosyllabic and polysyllabic words were indistinguishable, conversely to those arising from normal-appearing cortex [54].

#### Diffusion Tensor Imaging (DTI)

Tumor invasion may interact with white-matter tracts in three distinct patterns: infiltration, disruption, and displacement [41]. In patients with glioma, DTI sequences demonstrate a global decreased in fractional anisotropy and decreased axial, mean and radial diffusivity in the ipsilateral hemisphere, compared to the contralateral hemisphere [55]. Interestingly, preoperative tracking in patients with a left-sided glioma highlighted that patients with symmetric or right-lateralized posterior segment of the arcuate fasciculi had no language impairments. These data suggests that right homologs of structural language-associated pathways at the subcortical level could be supportive for language functions [56]. In clinical practice, glioma-network interrogation using DTI has prognostic significance for patients in whom awake resection is indicated, and to help in surgical planning [57].The data presented above suggest that glioma induced network remodeling is influenced by tumour biology which ultimately influences onco-functional outcomes.

#### Navigated Transcranial Magnetic Stimulation

Whereas direct electrostimulation is the gold standard method to detect functional areas, navigated Transcranial Magnetic Stimulation (nTMS) represents a unique non-invasive technique. As imaging techniques, nTMS objectivated an ipsilateral recruitment with a spread of motor areas in the post-central gyrus but also in the superior and middle frontal gyri, depending on tumor location compared to motor cortex [58]. Two studies assessed motor cortex remodeling before and after glioma resection, with the same limitations as studies previously described [52, 53]. The centers of gravity were shifted from 12.3 ± 14.3 mm [59], 4.6 ± 0.8 mm on the mediolateral axis, and 8.7 ± 1.5 mm on the anteroposterior axis [60]. Complete postoperative motor recovery was observed exclusively in patients with cortical remodeling [59]. nTMS targeting the right hemisphere induced language disturbances in right-handed patients with left-sided gliomas but not in healthy controls, confirming an underlying bilateral remodeling of the language network [57].

### Direct Electrostimulation

Causal evidence supporting glioma-induced cortical function remodeling has been demonstrated using direct electrical stimulation mapping. In patients who underwent repeated stimulation mappings during glioma resection, of 22 initially identified eloquent sites, 13 (59.1%) remained positive, while 9 (40.9%) had become negative although no neurological impairment was noted. These findings suggest that neurological function may be preserved through neural circuit remodeling or activation of latent functional pathways. Patients in who cortical function was lost were noted to have a smaller tumour volume compared to other patients but there were no differences regarding demographical, pathological or treatment-related features [61]. In another series of 42 patients who underwent repeated cortical and subcortical mappings during the awake resection of a glioma, patients with high-level plasticity (displacement of ≥ 2 eloquent sites) and low-level plasticity (displacement < 2 eloquent sites) were distinguished. In the high-level plasticity group, various rates of eloquent sites were gained or lost, including displacement of primary motor sites [62]. While initially thought to be static, cortical plasticity of primary sensorimotor sites has been reported with gained or lost function without corresponding neurologic impairments in 32/51 (62.7%) of stimulated sites [63]. These data refine the current understanding of glioma-induced modifications in neurocognitive processing and could guide new strategies including neuromodulation [54].

## Conclusion

Whereas functional imaging and per-operative acquisitions have defined patterns of malignant circuit remodeling at a macroscopic level, various in vitro and animal models have deciphered the complex bidirectional crosstalk between neurons and glioma cells at a microscopic level, leading to the emergence of new potential therapeutic targets. Future challenges will be to better understand how tumor genetics modulate neuron-glioma interactions and to integrate these data to design new therapeutic options likely to improve both oncological and functional outcomes.

## Data Availability

No datasets were generated or analysed during the current study.
